# Functional MRI of music emotion processing in frontotemporal dementia

**DOI:** 10.1111/nyas.12620

**Published:** 2015-03-13

**Authors:** Jennifer L Agustus, Colin J Mahoney, Laura E Downey, Rohani Omar, Miriam Cohen, Mark J White, Sophie K Scott, Laura Mancini, Jason D Warren

**Affiliations:** 1 Dementia Research Centre, UCL Institute of Neurology, University College LondonLondon, United Kingdom; 2 Neuroradiological Academic Unit, Department of Brain Repair and Rehabilitation, UCL Institute of Neurology, University College LondonLondon, United Kingdom; 3 Lysholm Department of Neuroradiology, National Hospital for Neurology and NeurosurgeryLondon, United Kingdom; 4 Institute of Cognitive Neuroscience, University College LondonLondon, United Kingdom

**Keywords:** music, voice, emotion, fMRI, bvFTD, musicophilia

## Abstract

Frontotemporal dementia is an important neurodegenerative disorder of younger life led by profound emotional and social dysfunction. Here we used fMRI to assess brain mechanisms of music emotion processing in a cohort of patients with frontotemporal dementia (*n* = 15) in relation to healthy age-matched individuals (*n* = 11). In a passive-listening paradigm, we manipulated levels of emotion processing in simple arpeggio chords (mode versus dissonance) and emotion modality (music versus human emotional vocalizations). A complex profile of disease-associated functional alterations was identified with separable signatures of musical mode, emotion level, and emotion modality within a common, distributed brain network, including posterior and anterior superior temporal and inferior frontal cortices and dorsal brainstem effector nuclei. Separable functional signatures were identified post-hoc in patients with and without abnormal craving for music (musicophilia): a model for specific abnormal emotional behaviors in frontotemporal dementia. Our findings indicate the potential of music to delineate neural mechanisms of altered emotion processing in dementias, with implications for future disease tracking and therapeutic strategies.

## Introduction

Music is an exceptionally emotionally rich and engaging sensory stimulus. Cognitive neuropsychology and functional neuroimaging studies in the healthy brain have shown that the neural mechanisms involved in analyzing music are intimately linked to the machinery of pleasure and reward.^1^,^2^ Emotion in music is processed by a complex distributed brain network architecture, including salience and evaluation systems in the insula, amygdala, and orbitofrontal cortex projecting to mesolimbic and subcortical dopaminergic pathways.^1^–^6^ More elemental affective attributes of dissonance and pleasantness, cognitive labeling of musical emotions, attribution of mental states to music, and appreciation of musical structure are likely to represent at least partially separable dimensions of music emotion coding.^2^–^5^,^7^,^8^ Taken together, this evidence suggests that the essentially abstract phenomenon of music may have had a neurobiological role during human evolution, perhaps by engaging a neural puzzle-solving algorithm that facilitates decoding of emotional mental states.^9^ This formulation underlines the social function of music, a key theme in contemporary neuroscience accounts.^2^,^9^

Various brain disorders have been found to impair music processing and, more particularly, perception and understanding of emotion in music.^9^–^14^ Within the neurodegenerative disease spectrum, the behavioral variant of frontotemporal dementia (bvFTD) produces striking deficits of music emotion processing as part of a wider syndrome of selective brain atrophy (chiefly affecting the frontal and temporal lobes) with impaired emotional understanding and responsiveness and profound disruption of interpersonal conduct.^15^–^19^ These patients exhibit a paradigmatic acquired disorder of social cognition that frequently spares general intellect; this creates challenges as well as opportunities. Clinically, bvFTD is liable to be misdiagnosed as a psychiatric disorder, and objective tools for characterizing abnormalities of emotional behavior remain very limited, although neurobiologically, bvFTD offers a unique window on critical brain network processes that support complex (particularly social) behaviors. Music is therefore especially attractive as a novel probe of disordered emotional and social signal processing in bvFTD: it promises to illuminate dysfunctional brain architecture (indeed, the study of bvFTD has already informed theoretical models of music biology^9^) and could potentially provide sensitive markers of brain dysfunction to aid clinical diagnosis and monitoring. Previous work on music emotion processing in bvFTD has focused largely on assessing recognition of emotions in musical pieces and correlation with structural neuroimaging of regional brain atrophy.^16^–^18^ Such approaches have several important limitations. The use of familiar music as an emotion carrier and procedures that depend on verbal labeling are potentially confounded by semantic impairment, which frequently accompanies bvFTD; meanwhile, structural neuroimaging techniques are essentially associational and cannot examine underlying dysfunctional brain mechanisms.

Here, we assessed brain mechanisms of music emotion processing in bvFTD directly, using functional MRI (fMRI) and a passive listening paradigm that manipulated two levels of emotion analysis—musical mode and dissonance—in simple chord sequences. This paradigm was well suited to address music emotion processing in patients with bvFTD on both behavioral and neuroanatomical grounds. Although musical modes, chords, and dissonance have well-established emotional resonances,^20^–^23^ these features lack the specific semantic associations of familiar melodies, particularly for nonmusicians.

The dimensions of consonance–dissonance and major–minor mode are likely to access different levels of music emotion representation. Although dissonance is a fundamentally aversive feature even for normal human infants,^24^ the processing of musical mode is likely to be strongly influenced by musical acculturation and cognitive set.^23^ However, these musical attributes have been shown to have robust brain substrates in normal human functional imaging and electrophysiological studies^25^–^27^ and differential vulnerability to focal brain insults^10^,^13^ in nonmusicians as well as trained musicians. Processing of musical mode has been shown to engage a distributed brain network, including prefrontal, anteromedial temporal, limbic, and brainstem areas,^20^,^25^,^28^,^29^ whereas processing of dissonance engages an overlapping anteromedial temporal, limbic, retrosplenial, and brainstem network.^4^,^25^ In this study, we compared music emotion processing with another domain of auditory emotion processing—human vocalizations—which is also vulnerable in bvFTD.^16^,^30^,^31^ We hypothesized that patients with bvFTD would show functional alterations of brain networks mediating musical mode and dissonance and vocal emotion processing relative to healthy older individuals and further that these dimensions of auditory emotion coding would have separable disease signatures within core corticolimbic circuitry that is targeted by the pathological process in bvFTD.^32^

## Methods

### Participants

Fifteen consecutive patients (three female; mean age 64 ± 8.2 (SD) years) fulfilling consensus diagnostic criteria for bvFTD^15^ and 11 healthy age-matched individuals (three female; mean age 64.0 ± 7.7 years) with no history of neurological or psychiatric disorders participated. No participant had a clinical history of hearing loss; none was a professional musician, and the patient and healthy control groups had similar musical backgrounds (years of formal musical training). Participant group demographic, clinical, and general neuropsychological data are summarized in Table S1. All patients had general neuropsychological assessments in keeping with their syndromic diagnosis and supportive structural brain MRI evidence of frontal and/or temporal lobe atrophy with no significant cerebrovascular burden. The bvFTD cohort was stratified post-hoc according to whether patients did or did not exhibit abnormal craving for music (musicophilia). This was defined operationally on the basis of a structured questionnaire^33^ as compulsive listening to music (typically, a small fixed repertoire of popular songs or classical pieces) for >10 h per week, where this represented a definite increase compared with premorbid levels. The patient subgroup with musicophilia (*n* = 6) had a mean age of 63 ± 8.1 years and symptom duration of 8.3 ± 5.2 years, similar to the patient subgroup without musicophilia (*n* = 9; age 64 ± 8.7 years; symptoms 8.9 ± 6.1 years). All participants gave informed consent in accordance with the Declaration of Helsinki.

### Experimental stimuli and conditions

In creating the fMRI paradigm, we adopted a subtractive design intended to isolate the effects of variation in emotional attributes of interest against matched baseline conditions with fixed attributes and thereby to identify brain regions specifically processing the relevant attribute without requiring an in-scanner task. Musical stimuli were based on four-note arpeggio chords (C major, G major, A minor, E minor) and their dissonant versions, synthesized as digital wave files with string instrument timbre using Sibelius® software (http://www.sibelius.com). Chords were concatenated to create sequences in which the identity of adjacent chords always varied but the sequence as a whole could be either constant in mode (major or minor) or interleaving major and minor modes and either uniformly consonant or dissonant or interleaving consonant (minor mode) and dissonant chords. In addition, wave files of human male and female nonverbal emotional vocalizations (laughter or crying^34^) were concatenated to create sequences in which the gender for consecutive vocal segments always varied but the sequence as a whole could be either constant in emotion (laughter or crying) or interleave laughter and crying. All individual sequence elements (chords or vocalizations) were 1.5 seconds in duration with fixed mean intensity; each sequence comprised five elements (overall duration 7.5 seconds) in ABABA configuration. This experimental design yielded six conditions (music fixed mode consonant, MFC; music fixed dissonant, MFD; music changing mode, MCM; music changing dissonance, MCD; vocal fixed emotion, VF; vocal changing emotion, VC) plus an additional silence (rest) condition. Further details about stimuli and conditions are in Table S2.

### Scanning protocol

During scanning, 16 trials in each fixed emotion condition, 32 trials in each changing emotion condition, and 10 silence trials (186 trials in total, in two consecutive runs) were presented in pseudorandomized, miniblocked order at a comfortable listening level (at least 70 dB) binaurally via pneumatic headphones embedded in ear-defenders (Esys fMRI system, Invivo Corporation, Orlando, FL, USA). Participants were asked to listen to the sounds with their eyes lightly closed, with no output task. In-house software written in Python (http://www.python.org) was used to integrate stimulus delivery with the scanner controls.

Brain images were acquired on a 3T TIM Trio whole-body MRI scanner (Siemens Healthcare, Erlangen, Germany). Functional echoplanar images were obtained in a sparse-sampling protocol with 8-second interscan pauses during which auditory stimuli were delivered; a B0 field-map for subsequent inhomogeneity correction and a volumetric MPRAGE structural sequence were also acquired for each participant. Further details about image acquisition can be found in Supporting Information.

### Postscan behavioral assessments

After scanning, participants performed a two-alternative forced choice (same/different) 1-back psychophysical task to assess their perception of the musical stimuli (further details in Supporting Information). Results were analyzed using Stata 12.1® (StataCorp LP, College Station, TX, USA): a linear-regression model incorporated accuracy scores for all participants with between-group effects of interest and nuisance covariates of age, gender, and reverse digit span (a standard index of auditory working memory) thresholded at *P* < 0.05.

### Analysis of fMRI data

The fMRI data were analyzed using statistical parametric mapping software (SPM8; http://www.fil.ion.ucl.ac.uk/spm). Functional scans for each participant were realigned to the first image, unwarped using field-map for distortions correction, and coregistered with the structural brain image. Structural images for all participants were then segmented into component tissue types and entered into the DARTEL toolbox^35^ to create a study-specific group mean template brain image for rendering statistical parametric maps. Functional and structural images were normalized to the structural template image aligned in MNI standard stereotactic space and smoothed with a 6-mm full-width-at-half-maximum smoothing kernel.

Processed fMRI data from both scanning runs were entered separately for each participant into first-level design matrices that modeled auditory conditions and rest as separate regressors comprising boxcars of one trial duration convolved with the canonical hemodynamic response function; six regressors of noninterest modeled head movements extracted during realignment. Experimental contrasts were constructed to assess the effects of auditory stimulation (all auditory conditions > rest), musical mode variation (MCM > MFC), musical dissonance variation (MCD > (MFC + MFD)), vocal emotion variation (VC > VF), music emotion level (the interaction of changing musical mode and changing musical dissonance: (MCM > MFC) > (MCD > MFD)); and music-specific emotion (the interaction of changing musical mode and changing vocal emotion: (MCM > MFC) > (VC > VF)). First-level contrast images of interest for each participant were then entered into second-level between-group two-sample *t*-tests (controls versus bvFTD) with covariates of age and gender; a similar between-subgroup second-level analysis was conducted post-hoc to compare patients with and without musicophilia. Contrasts were thresholded at peak statistical significance criterion *P* < 0.05 after family-wise error (FWE) correction for multiple voxel-wise comparisons over the whole brain (FWE whole brain) or within the prespecified anatomical region of interest (FWE small volume). Anatomical small volumes were derived from previous functional neuroimaging and lesion work^4^,^10^,^13^,^20^,^25^,^28^,^29^ and defined using the Harvard brain maps (http://www.fmrib.ox.ac.uk/fsl), comprising auditory association cortex in planum temporale and posterior superior temporal gyrus, anteromedial temporal lobe including amygdala and hippocampus, and inferior frontal lobe. To better interpret significant interactions, peak voxel β parameter estimates were extracted for each component condition and participant and analyzed post-hoc using paired within-group *t*-tests in Stata 12.1®.

## Results

### Functional MRI findings

**Figure 1:**
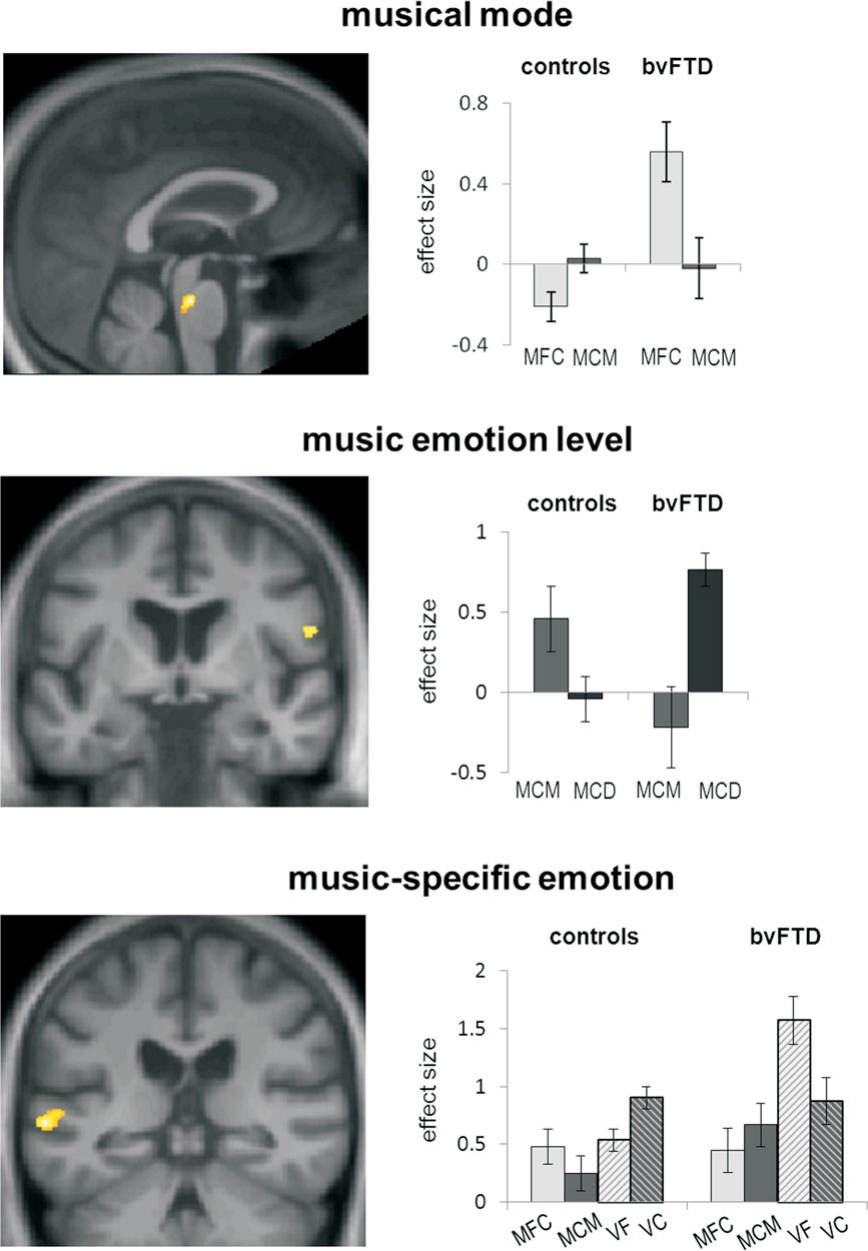
Statistical parametric maps (SPMs; left panels) of significant between-group contrasts and effect sizes (group mean ± SD peak voxel *β* parameter estimates; right panels) in key music emotion conditions for behavioral variant frontotemporal dementia (bvFTD) and healthy control groups. SPMs are rendered on a study-specific group anatomical image in MNI space (threshold *P* < 0.001 uncorrected over whole brain for display; see also Table1). Contrasts were based on interactions as follows: musical mode, ((MCM > MFC) × (controls > bvFTD)); music emotion level, ((MCD > MCM) × (control < bvFTD)); music-specific emotion, ((MCM > MFC) × (VC > VF) × (controls < bvFTD)). MFC, music fixed mode consonant; MCM, music changing mode; MCD, music changing dissonance; VC, vocal changing emotion; VF, vocal fixed emotion.

Significant activations for the experimental contrasts of interest are summarized in Table1, and statistical parametric maps and condition mean parameter estimates are displayed in Figure1. Auditory stimulation per se in both patients and healthy older individuals produced bilateral activation of auditory cortex including Heschl's gyrus, planum temporale, and superior temporal gyrus (*P* < 0.05, FWE whole brain), with no significant between-group differences.

**Table 1:** Summary of significant contrasts and regions of activation for patients vs. healthy controls^a^

					Peak (mm)				
Contrast		Area	Side	Cluster size (voxels)	*x*	*y*	*z*	*P* value	*Z-* score
Musical mode	bvFTD > controls	Dorsal brainstem	–	306	1	−29	−24	**0.037**	5.12
Music emotion level	Controls > bvFTD	PT	L	51	−65	−29	0	0.013	3.55
	bvFTD > controls	IFG	R	90	55	12	11	0.028	3.77
Vocal emotion	Controls > bvFTD	Anterior STG	L	20	−52	−10	−11	0.021	3.53
		Posterior STG	L	100	−59	33	1	0.024	3.63
Music-specific emotion	bvFTD > controls	Posterior STG	L	292	−61	−31	0	0.001	4.67

a Significant interactions of key emotion-processing contrasts with group are shown; boldface indicates contrast significant at *P* < 0.05 after FWE correction for multiple voxel-wise comparisons over the whole brain; other contrasts significant at *P* < 0.05 after FWE correction for multiple comparisons over prespecified anatomical regions of interest. Statistics and coordinates (in MNI space) for local maxima of activation are shown. Condition contrasts were defined as follows (see text for condition labels): musical mode (MCM > MFC); music emotion level ((MCM > MFC) > (MCD > MFD)); vocal emotion (VC > VF); music-specific emotion ((MCM > MFC) > (VC > VF)). bvFTD, behavioral variant of frontotemporal dementia; IFG, inferior frontal gyrus; PT, planum temporale; STG, superior temporal gyrus.

Musical mode variation produced greater activation in dorsal brainstem (in the region of the raphe nuclei and locus coeruleus) in the healthy control group than in the bvFTD group ((MCM > MFC) × (controls > bvFTD), *P* < 0.05 FWE whole brain)); post-hoc analysis of *β* parameter estimates showed that this interaction was driven by a crossover interaction, whereby changing mode enhanced activity in controls (*t*_10_ = −3.31, *P* = 0.008) and reduced activity in patients (*t*_10_ = 6.68, *P* < 0.001) compared to fixed mode. Although musical dissonance variation produced significant activation in the left amygdala and right inferior frontal cortex in the bvFTD group, no significant differences with respect to healthy controls were observed for this contrast. However, there was a significant effect of music emotion level in the left planum temporale and right inferior frontal gyrus in the bvFTD group compared to the healthy control group ((MCD > MCM) × (control < bvFTD), *P* < 0.05 FWE small volume)); post-hoc analysis of *β* parameter estimates revealed that this interaction was driven by patient group effects of greater responses to musical mode than dissonance variation in left planum temporale (*t*_14_ = −2.22, *P* = 0.04) with no effect in controls, and the reverse pattern in right inferior frontal gyrus (*t*_14_ = 4.24, *P* < 0.001) with the opposite effect in controls (*t*_10_ = −2.34, *P* = 0.035).

Vocal emotion variation produced greater activation in the left anterior and posterior superior temporal sulcus in the healthy control group than in the bvFTD group ((VC > VF) × (controls > bvFTD), *P* < 0.05 FWE small volume)); post-hoc analysis of *β* parameter estimates showed that this effect was driven by a crossover interaction whereby changing vocal emotion enhanced activity in controls and reduced activity in patients compared to fixed vocal emotion for both anterior (controls: *t*_10_ = 2.58, *P* = 0.027; bvFTD: *t*_14_ = −2.69, *P* = 0.018) and posterior (controls: *t*_10_ = 2.44, *P* = 0.035; bvFTD: *t*_14_ = −3.72, *P* = 0.002) areas in superior temporal sulcus. In addition, music-specific emotional responses in an overlapping region of left posterior superior temporal sulcus were greater in the bvFTD group than in the healthy control group ((MCM > MFC) × (VC > VF) × (controls < bvFTD), *P* < 0.05, FWE small volume)); post-hoc analysis of *β* parameter estimates confirmed that this three-way interaction effect was driven by attenuated responses to musical mode variation (*t*_10_ = −2.43, *P* = 0.035) and enhanced responses to vocal emotion variation (*t*_10_ = 2.40, *P* = 0.037) in controls and the reverse response pattern in patients. No other significant effects were found at the prescribed threshold.

The post-hoc analysis comparing patient subgroups with and without musicophilia (Table S3) revealed separable activation profiles: the musicophilic subgroup showed greater activation of anterior superior temporal cortex for the effect of auditory stimulation and planum temporale for musical dissonance variation, whereas the nonmusicophilic subgroup showed greater activation of the temporal pole for the effect of musical dissonance variation and orbitofrontal cortex and amygdala for music-specific emotional responses (all *P* < 0.05, FWE small volume).

### Postscan behavioral findings

Both bvFTD patients and healthy control participants performed significantly better than chance (proportion correct 0.5) in the postscan task assessing their discrimination of the musical stimuli (*t*-test results on proportion correct: controls, mean ± SD = 0.90 ± 0.10, *t*_10_ = 13.44, *P* < 0.001; bvFTD, mean ± SD = 0.78 ± 0.13, *t*_12_ = 7.68, *P* < 0.001). Performance accuracy on the task did not significantly differ between the patient and healthy control groups (*P* = 0.2).

## Discussion

Here we have demonstrated functional neuroana-tomical signatures of disease-associated alterations of musical and vocal emotion coding in a canonical dementia syndrome, bvFTD, relative to healthy older individuals. The present findings are in line with previous behavioral and structural neuroanatomical evidence for altered processing of emotion in music and other modalities in bvFTD,^16^–^18^ but in contrast to that previous work, we delineate functional brain network changes directly. The brain regions identified here as loci of altered processing in bvFTD include hubs within a distributed neural network that has been previously implicated both in the pathogenesis of bvFTD^19^,^36^ and in the analysis of music and other emotional sounds.^1^,^2^,^9^ The planum temporale and the auditory association cortex in the posterior superior temporal lobe are likely to mediate an early parsing of the auditory scene that disambiguates salient auditory sources from the acoustic background and initiates the process of auditory object identification,^37^ processes relevant to the analysis of both melodies and voices. Furthermore, these areas communicate with cross-modal and reward circuitry involved in evaluating the emotional significance of sounds.^38^–^40^ Inferior frontal cortex has been implicated in processing mode and tonality in music,^28^,^41^ and anterior superior temporal cortex has been implicated in processing emotional and other attributes of human vocalizations.^42^

Processing of musical mode here produced robust differential activation of dorsal brainstem in the region of the midline raphe nuclei and locus coeruleus in patients with bvFTD relative to healthy older individuals. A broadly similar association has been reported previously in the healthy brain during passive listening to musical chord changes^25^ and underpinning the modulatory effect of music on pain perception.^43^ These brainstem nuclei are major effector hubs in coordinating primitive arousal and emotional responses to sensory stimulation via widespread ascending and descending serotonergic and noradrenergic pathways. Furthermore, they have been proposed as key output sites mediating dysfunctional large-scale network responses to salient stimuli in bvFTD.^36^

Although care is needed in interpreting the post-hoc analysis comparing patients with and without musicophilia based on small case numbers, there was evidence of a separation of music emotion-processing mechanisms underpinning these music behavior phenotypes. Musicophilia was associated with relatively enhanced activation of auditory association cortex, whereas the absence of musicophlia was associated with relatively enhanced activation of orbitofrontal and anteromesial temporal lobe, including the amygdala. This differentiation might reflect relative processing biases toward musical pattern analysis versus evaluation (and, potentially, censoring) of musical behavioral responses, respectively, a formulation in line with recent general models of music emotion analysis.^1^,^2^,^9^ However, further work is required to substantiate this conjecture.

Taken together, the present findings reveal a complex profile of functional alterations linked to this neurodegenerative syndrome. Relative to healthy individuals, bvFTD was associated with bidirectional activity shifts and separable regional functional signatures of emotion modality (music versus nonverbal vocal) and processing level (musical mode versus dissonance) within a common distributed frontotemporosubcortical network. This complexity is in line with functional signatures demonstrated previously for semantic analysis of nonverbal sounds in another canonical neurodegenerative syndrome, semantic dementia.^44^ It is of interest that neither auditory stimulation per se nor the processing of musical dissonance showed a significant disease effect here. This suggests that the coding of more complex emotion information (such as musical mode) may be relatively more vulnerable to the neurodegenerative process in bvFTD.

This study has several limitations that suggest directions for future work. Case numbers were relatively small, and findings require corroboration in larger cohorts; this is an issue of particular relevance in bvFTD, which is a highly pathologically heterogeneous syndrome underpinned by accumulation of diverse pathogenic proteins with potentially distinct patterns of network disintegration.^45^ It will also be important to assess bvFTD alongside Alzheimer's disease and other dementia syndromes in order to further identify the disease specificity of the signatures identified. Our paradigm was based on passive listening to generic emotional stimuli with relatively simple structure. In daily life, however, much of the emotional impact of music is carried by more complex structures such as melodies and rhythms, often with specific semantic associations. The effects of neurodegenerative disease on the brain mechanisms that process these more familiar musical entities remain to be defined. Furthermore, although postscan behavioral testing here established that patients were able to perceive stimulus changes comparably to healthy individuals, the extent to which task demands may modulate the functional neuroanatomy of music emotion processing remains unclear. Notwithstanding these caveats, the present findings provide a prima facie case for a more comprehensive functional neuroanatomical analysis of emotion processing in the dementias. It will be of particular interest to determine whether musical and social signal processing share functional brain circuitry in these diseases and whether, indeed, music might serve as a model for dysfunctional social brain mechanisms in bvFTD and other neurodegenerative syndromes^9^,^18^ and, furthermore, to identify substrates for specific musical behavior phenotypes.^33^ In addition to revealing disease mechanisms, music may constitute a sensitive biomarker of emotional dysfunction in neurodegenerative disease with implications for diagnosis and monitoring. More speculatively, the powerful neuromodulatory effect of music on behavior in brain disorders^33^,^43^ might have therapeutic potential in dementia syndromes if interventions are informed by valid pathophysiological models.

## Supporting Information

**Table S1**. Demographic and general neuropsychological data for participant groups **Table S2**. Experimental auditory stimuli and conditions **Table S3**. Significant contrasts and regions of activation for musicophilic versus nonmusicophilic patients
