# Supplementary material for: Functional MRI of music emotion processing in frontotemporal dementia
Source: Ann N Y Acad Sci. 2015 Mar 13;1337(1):232–40. doi: 10.1111/nyas.12620 (PMC4402026; doi:10.1111/nyas.12620)
Supplement: Supplementary file 1 — Table S1. Demographic and general neuropsychological data for participant groups Table S2. Experimental auditory stimuli and conditions Table S3. Significant contrasts and regions of activation for musicophilic versus nonmusicophilic patients [file nyas1337-0232-sd1.docx]

**SUPPLEMENTARY MATERIAL**

**Functional MRI of musical emotion processing in frontotemporal dementia,**

by JL Agustus et al

**Brain image acquisition details**

Each functional scanning run comprised 97 gradient-echo echo-planar image (GE-EPI) volumes of 48 oblique transverse slices with slice thickness 2mm, inter-slice gap 1mm, 2 x 2 mm in-plane resolution and -30^o^ T>C tilt (TR/TE = 11000/30ms; echo spacing = 0.69ms; matrix size = 96 x 96 pixels; FoV = 192 x 192mm; GRAPPA factor 2 in anterior-posterior phase encoding direction). The first four EPI volumes were discarded to allow for equilibration of T1 longitudinal magnetisation.

A B0 field-map was acquired (TR = 688ms; TE1/TE2 = 4.92/7.38ms, 3x3x3mm resolution, no inter-slice gap; matrix size = 80 x 80 pixels; FoV = 192 x 192mm; phase encoding direction = anterior-posterior) to allow unwarping of any B0 field inhomogeneity induced geometric distortions on the EPI data. A structural MPRAGE sequence protocol with 32 channel RF receiver head coil was used to acquire a 1.1mm isotropic volumetric brain image (TR = 2.2s; TE = 2.9ms; matrix size = 256 x 256 pixels; FoV = 282 x 282 mm).

**Post-scan behavioural test details**

Participants were presented with paired chords derived from the experimental stimulus set via a notebook computer; twenty-four trials (chord pairs) were presented in randomised order, such that the members of each pair were either identical or differed in mode or dissonance. Prior to behavioural testing, participants were familiarised with the task and were told that a difference between the musical sounds would correspond to their being ‘in tune’ versus ‘out of tune’ (consonant – dissonant) or ‘happy’ versus ‘sad’ (major – minor mode). During testing, the task on each trial was to indicate whether the two sounds were ‘the same or different’. No feedback about performance was given and no time limits were imposed. The proportion of correct responses was calculated as a measure of task performance accuracy. This post-scan behavioural test was not undertaken by two patients and was terminated early in another patient.

**Table S1. Demographic and general neuropsychological data for participant groups**

| **Characteristic** | **bvFTD** | **Healthy controls** |
| --- | --- | --- |
| **General** | | |
| No. (F:M) | 15 (3:12) | 11 (3:8) |
| Handedness | 13R, 2L | 10R, 1L |
| Age (years) | 64 (8.2) | 64 (7.7) |
| Education (years) | 15 (3.5) | 17 (1.2) |
| Musical training (years) | 3.9 (4.3) | 3.3 (4.5) |
| Current music listening (hrs /wk) | 14.3 (14.3)* | 9.4 (8.3) |
| Symptom duration (years) | 8.6 (5.5) | N/A |
| MMSE (/30) | **26 (3.2)** | **30 (0.4)** |
| **Neuropsychological** | | |
| ***General cognitive*** | | |
| IQ Verbal | **90 (20)** | **121 (10)** |
| IQ Performance | **96 (18)** | **121 (11)** |
| ***Episodic and short term memory*** | | |
| RMT Faces (/50) | **34 (9.3)** | **44 (5.8)** |
| RMT Words (/50) | **36 (4.6)** | **47 (3.1)** |
| DS forward total (/12) | 8 (2.5) | 9 (1.7) |
| DS backward total (/12) | 6 (2.4) | 7 (1.4) |
| ***Language and short term memory*** | | |
| BPVS (/150) | **128 (20)** | **147 (2.2)** |
| GNT (/30) | **11 (9.2)** | **26 (4.2)** |
| Reading: NART (/50) | **33 (12)** | **42 (3.9)** |
| ***Posterior cortical*** | | |
| Arithmetic (/24) | 8 (9.3) | 15 (3.9) |
| VOSP (/20) | 17 (3.2) | 19 (1.2) |
| ***Executive and social cognition*** | | |
| D-KEFS Stroop: Colour (s) | **38 (7.0)** | **30 (4.0)** |
| D-KEFS Stroop: Word (s) | 24 (3.9) | 21 (4.5) |
| D-KEFS Stroop: Inhibition (s) | **79 (23)** | **56 (11)** |
| TASIT Emotion (total) | **8 (1.9)** | **11 (2.4)** |
| TASIT Social Inference (total) | **21 (7.5**) | **32 (2.4)** |

Mean (standard deviation) data are shown unless otherwise indicated; significant group differences (p<0.05, two-sample between-group t-tests) are in bold. *musicophilic subgroup 21.4(14.5) hours/week, non-musicophilic subgroup 3.5(2.7) hours/week; Arithmetic, Graded Difficulty Arithmetic test (Jackson and Warrington, 1986, Cortex 22:611–620); BPVS, British Picture Vocabulary Scale (Dunn et al., 1982, NFER-Nelson, Windsor); bvFTD, behavioural variant frontotemporal dementia; D-KEFS, Delis-Kaplan Executive Function System colour-word inhibition Stroop task; DS, Digit Span test from Wechsler Memory Scale-Revised (Wechsler, 1987, The Psychological Corporation, San Antonio); GNT, Graded Naming Test (McKenna and Warrington, 1983, NFER-Nelson, Windsor); IQ, verbal/performance intelligence quotient from Wechsler Abbreviated Scale of Intelligence (Wechsler, 1999, The Psychological Corporation, San Antonio); MMSE, Mini-Mental State Examination (Folstein et al., 1975, J. Psychiatr. Res. 12: 189–198); N/A, not applicable; RMT, Recognition Memory Test (Warrington, 1984, NFER-Nelson, Windsor); TASIT, The Awareness of Social Inference Test (McDonald et al., 2003, J Head Trauma Rehabil. 18: 219-238); VOSP, Object Decision Test from Visual Object and Space Perception Battery (Warrington and James, 1991, Thames Valley Test Company, Bury St Edmunds).

**Table S2. Experimental auditory stimuli and conditions**

| **Code** | **Experimental auditory condition** | **No.**  **trials** | **Seq**  **ex** | **Stimulus A**  **seq pos. 1^st^, 3^rd^, 5^th^** | **Stimulus B**  **seq. pos. 2^nd^, 4^th^** |
| --- | --- | --- | --- | --- | --- |
| **MFC** | Music fixed mode consonant:  major mode | 16 | 1.  2. | C major chord  G major chord | G major  C major |
|  | Music fixed mode consonant:  minor mode | 16 | 1.  2. | A minor  E minor | E minor  A minor |
| **MFD** | Music fixed dissonant | 16 | 1.  2. | A minor dissonant  E minor dissonant | E minor dissonant  A minor dissonant |
| **MCM** | Music changing mode | 32 | 1.  2.  3.  4. | A minor  C major  E minor  G major | C major  A minor  G major  E minor |
| **MCD** | Music changing dissonance | 32 | 1.  2.  3.  4. | A minor dissonant  A minor  E minor dissonant  E minor | A minor  A minor dissonant  E minor  E minor dissonant |
| **VF** | Vocal fixed emotion:  happiness | 16 | 1.  2. | female laughing  male laughing | male laughing  female laughing |
|  | Vocal fixed emotion:  sadness | 16 | 1.  2. | female crying  male crying | male crying  female crying |
| **VC** | Vocal changing emotion | 32 | 1.  2.  3.  4. | female laughing  male crying  female crying  male laughing | male crying  female laughing  male laughing  female crying |

Each stimulus sequence contained two 1.5 second sound elements (A, B) that were concatenated in alternating sequence positions (seq. pos.; i.e. ABABA) to create a 7.5 second trial sequence. Adjacent chords were in proximity on the circle of fifths according to Western musical theory, in order to minimise concomitant pitch variations. Two trial sequence exemplars (seq. ex.) were presented in each ‘fixed’ emotion condition and four in each ‘changing’ emotion condition; an additional silence (rest) condition (10 trials) was also included.

**Table S3. Significant contrasts and regions of activation for musicophilic vs non-musicophilic patients**

| **Contrast** | | **Area** | **Side** | **Cluster size** (voxels) | **Peak (mm)** | | | **P value** | **z-score** |
| --- | --- | --- | --- | --- | --- | --- | --- | --- | --- |
|  |  |  |  |  | x | y | z |  |  |
| All sound | M > NM | Anterior STG | R | 40 | 52 | 0 | -19 | 0.008 | 4.31 |
| Musical dissonance | M> NM | PT | R | 44 | 55 | -21 | 10 | 0.023 | 3.86 |
|  | NM > M | Temporal pole | L | 39 | -53 | 18 | -13 | 0.009 | 4.64 |
| Music-specific emotion | M > NM | Anterior STG | L | 21 | -61 | 1 | -9 | 0.036 | 3.48 |
|  | NM > M | OFC | L | 67 | -9 | 49 | -8 | 0.019 | 4.21 |
|  |  | Anterior STG | L | 21 | -49 | 2 | -17 | 0.030 | 3.54 |
|  |  | Amygdala | L | 27 | -14 | -5 | -17 | 0.023 | 3.65 |

Significant interactions of key emotion processing contrasts with subgroup from post hoc analysis are shown; all contrasts significant p < 0.05 after FWE correction for multiple comparisons over pre-specified anatomical regions of interest and exceeding cluster size 20 voxels are included. Statistics and coordinates (in MNI space) for local maxima of activation are shown. Condition contrasts were defined as follows (see text for condition labels): all sound, (all auditory conditions > rest); musical dissonance, (MCD > [MFC+MFD]); music-specific emotion, ([MCM > MFC] > [VC > VF]). M, subgroup of patients with musicophilia; NM, subgroup of patients without musicophilia; OFC, orbitofrontal cortex; PT, planum temporale; STG, superior temporal gyrus.
